# Supplementary figures and images for: Buffalo bbu-miR-493-5p Promotes Myoblast Proliferation and Differentiation
Source: Animals (Basel). 2024 Feb 6;14(4):533. doi: 10.3390/ani14040533 (PMC10886120; doi:10.3390/ani14040533)

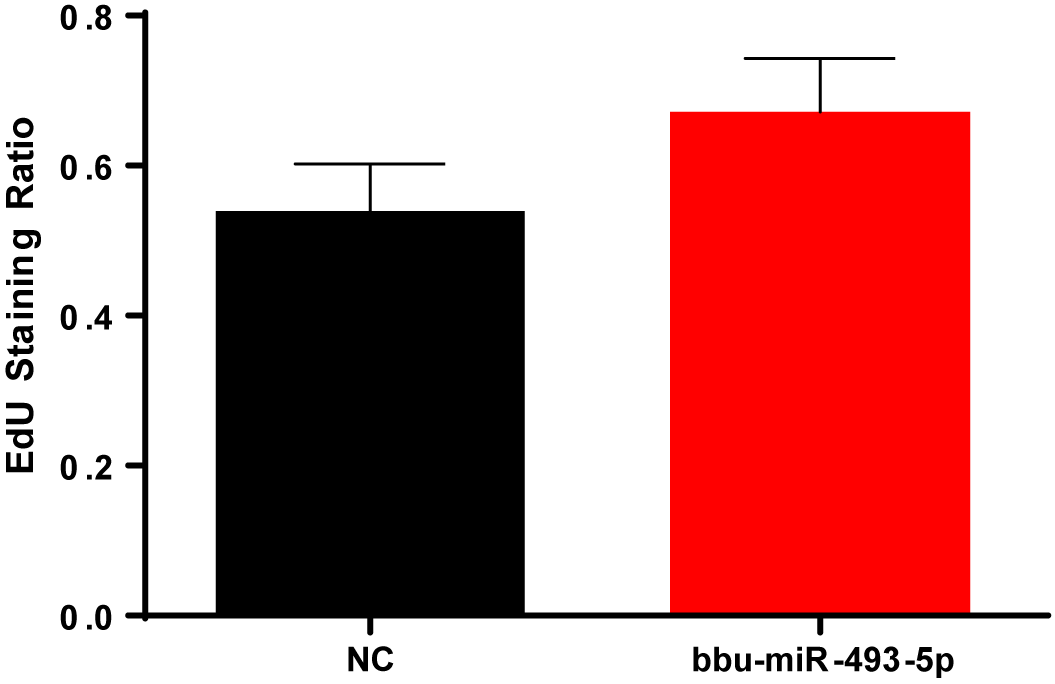

Supplement: Supplementary file 1 [file animals-14-00533-s001.zip › Supplementary Figure S1 EdU Statistical Analysis Chart.tif]
